# ﻿Four new species of the genus *Trilacuna* Tong & Li, 2007 (Araneae, Oonopidae) from China

**DOI:** 10.3897/zookeys.1219.138793

**Published:** 2024-12-06

**Authors:** Jimeng Ma, Qiang Chen, Yanfeng Tong

**Affiliations:** 1 Life Science College, Shenyang Normal University, Shenyang 110034, China Shenyang Normal University Shenyang China; 2 Experimental Teaching Center, Shenyang Normal University, Shenyang 110034, China Shenyang Normal University Shenyang China

**Keywords:** Distribution, goblin spiders, morphology, taxonomy

## Abstract

Four new species of the genus *Trilacuna* Tong & Li, 2007, *Trilacunaguangwu* Ma & Tong, **sp. nov.** (♂), *Trilacunaqingliangfeng* Ma & Tong, **sp. nov.** (♂♀), *Trilacunataoyuanyu* Ma & Tong, **sp. nov.** (♂) and *Trilacunayunmeng* Ma & Tong, **sp. nov.** (♂), are described from China. Descriptions, diagnoses, and photographs are provided.

## ﻿Introduction

*Trilacuna* Tong & Li, 2007 is a small genus in Oonopidae Simon, 1890 and currently comprises 44 species (WSC 2024). Known species are from East Asia (22 species in China and one in South Korea), Southeast Asia (15 species), South Asia (five species) and West Asia (one species) (Eichenberger and Kranz-Baltensperger 2011; Grismado et al. 2014; Malek-Hosseini et al. 2015; Seo 2017; Tong et al. 2024). In China, all the known species are recorded in southwestern China, of which seven from Chongqing, two are from Guizhou, and 13 are from Yunnan Province (Tong et al. 2019; Huang et al. 2020, 2021; Wang et al. 2021; Ma et al. 2023). Examination of oonopid specimens preserved in Hebei University (Baoding, China) found four new species of the genus *Trilacuna*, of which one species was collected from Anhui and Zhejiang Provinces, and three species were collected from Hebei, Fujian, and Sichuan Provinces, respectively. Until now, the record from Hebei is the northernmost record of this genus in the world. This work describes four new *Trilacuna* species from outside of southwestern China.

## ﻿Materials and methods

Specimens were examined using a Leica M205 C stereomicroscope. Fine details were studied under an Olympus BX51 compound microscope. Endogynes were cleared in lactic acid. Photomicroscope images were taken with a Canon EOS 750D zoom digital camera (24.2 megapixels) mounted on an Olympus BX51 compound microscope. Raw photos were first stacked with Helicon Focus v. 8.2.0 to get the composite images, which were then processed in Adobe Photoshop CC 2020. Scanning electron microscope images (SEM) were taken under high vacuum with a Hitachi S-4800 after critical point drying and gold-palladium coating. All measurements were taken using an Olympus BX51 compound microscope and are in millimeters. Taxonomic descriptions follow Tong et al. (2020). Type material is deposited in Shenyang Normal University (SYNU) in Shenyang, Liaoning Province, China (curator: Yanfeng Tong).

The following abbreviations are used in the text and figures: **ALE** = anterior lateral eyes; **ap** = apodemes; **as** = anterior sclerite; **bep** = basal ear-shaped projection; **bls** = brush-like structure; **glo** = globular structure; **lcb** = lateral curved branch; **mb** = medial branch; **mr** = membranous region; **PLE** = posterior lateral eyes; **PME** = posterior media eyes; **psp** = posterior spiracle; **sar** = sclerotized, recurved arches; **sdb** = slightly curved distal branch; **tls** = thorn-like structure; **tsc** = transverse bars.

## ﻿Taxonomy

### ﻿Family Oonopidae Simon, 1890

#### ﻿Genus Trilacuna

Taxon classificationAnimaliaAraneaeOonopidae

Tong & Li, 2007

81D578DA-DB58-5546-A4FA-D050F5906737

##### Type species.

*Trilacunarastrum* Tong & Li, 2007 from Yunnan, China.

##### Diagnosis.

See Tong et al. (2020).

##### Composition.

48 species, including four described here.

##### Distribution.

Iran to the Korean Peninsula and south to Sumatra.

#### ﻿ Trilacuna guangwu

Taxon classificationAnimaliaAraneaeOonopidae

Ma & Tong sp. nov.

38A192AE-E593-5BAA-AD31-0D6A46C88F99

https://zoobank.org/C85D9470-36D7-4DCF-9CD7-3DAF675BBB05

Figs 1 , 2 , 6C

##### Material examined.

***Holotype***: China • ♂ (SYNU-1179); Sichuan Province, Bazhong City, Nanjiang County, Guangwu Mountain, Taoyuan Scenic Area; 32°40'48"N, 106°48'12"E; 5.VIII.2014; F. Zhang leg.

##### Etymology.

The specific name is a noun in apposition taken from the type locality.

##### Diagnosis.

**Figure 1.**
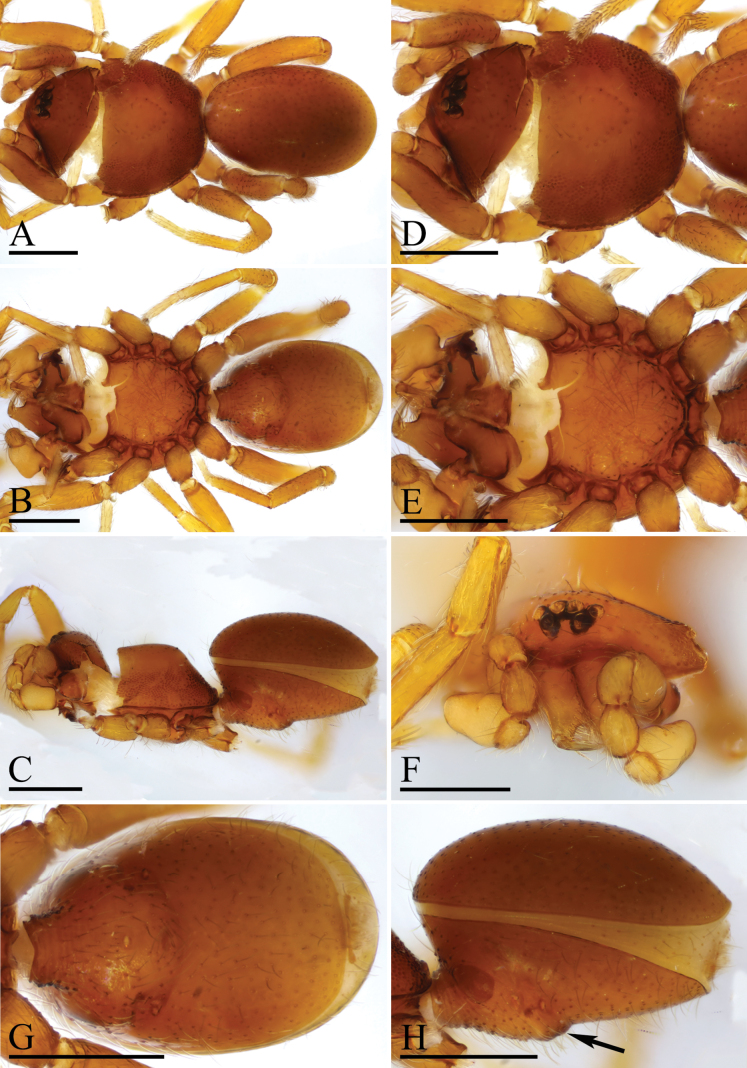
*Trilacunaguangwu* sp. nov., male holotype **A–C** habitus in dorsal, ventral and lateral views **D–F** prosoma in dorsal, ventral and anterior views **G–H** abdomen, ventral and lateral views, arrow shows the elevated epigastric region. Scale bars: 0.4 mm (**A–H**).

**Figure 2.**
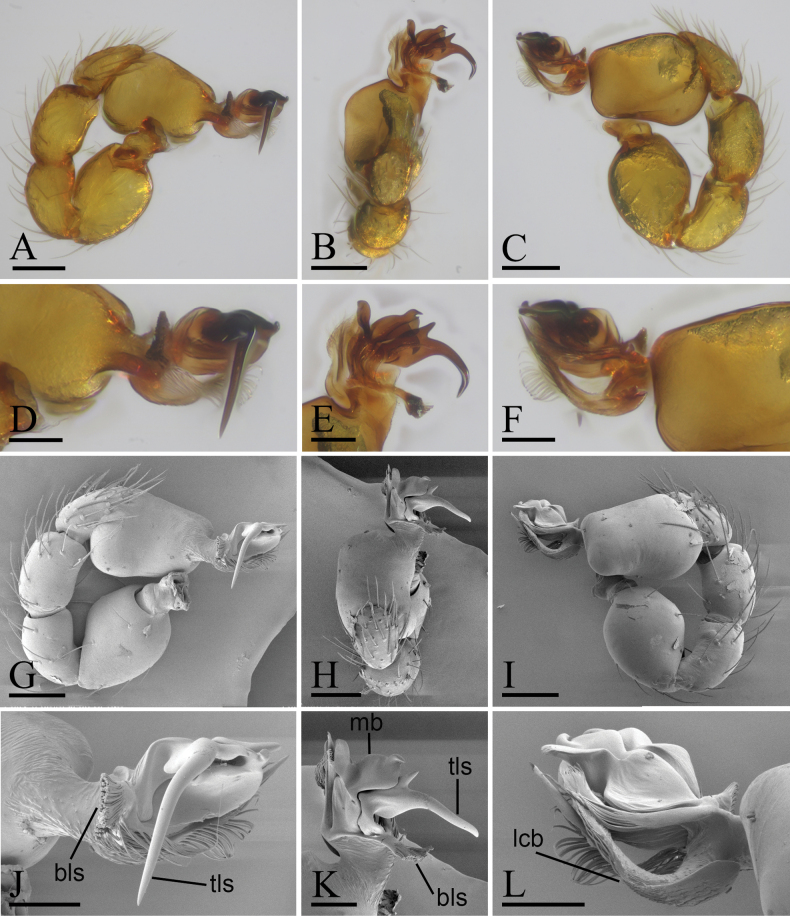
*Trilacunaguangwu* sp. nov., left male palp, **A–F** (light) and **G–L** (SEM) **A, G** prolateral view **B, H** dorsal view **C, I** retrolateral view **D, J** distal part of palpal bulb, prolateral view **E, K** distal part of palpal bulb, dorsal view **F, L** distal part of palpal bulb, retrolateral view. Abbreviations: bls = brush-like structure; lcb = lateral curved branch; mb = medial branch; tls = thorn-like structure. Scale bars: 0.1 mm (**A–C, G–I**); 0.05 mm (**D–F, J–L**).

The new species is similar to *Trilacunaangularis* Tong & Li, 2007, but can be distinguished by the large, thorn-like structure (tls) and brush-like structure (bls) of embolus system (Fig. 2D, E, J, K) vs lacking, but with several strongly sclerotized sclerites (Tong and Li 2007: fig. 15–18), and slightly elevated epigastric region (Fig. 1H) vs flat (Tong and Li 2007: fig. 14).

##### Description.

**Male** (Holotype). ***Body***: reddish brown, chelicerae and sternum lighter, legs yellow; habitus as in Fig. 1A–C; body length 2.05. ***Carapace***: 1.06 long, 0.81 wide; sides granulate, lateral margin rebordered (Fig. 1D). ***Eyes***: nearly equal sized; posterior eye row straight from above, procurved from front; ALE separated from edge of carapace by 1.4 diameters (Fig. 1D, F). ***Mouthparts***: chelicerae straight; labium rectangular, anterior margin deeply incised; endites slender, distally not branched, with a membranous region (Figs 1E, 6C). ***Sternum***: surface rugose (Fig. 1E). ***Abdomen***: 1.01 long, 0.65 wide; booklung covers ovoid, surface smooth; sperm pore situated at level of anterior spiracles; apodemes present, posterior spiracles connected by groove; epigastric region slightly elevated (Fig. 1G, H). ***Leg spination***: legs I-II: tibia: v2-2-2-2-0, metatarsus: v2-2-0. ***Palp***: orange; 0.66 long (0.21, 0.15, 0.15, 0.15); femur greatly enlarged (width/length = 0.74); bulb quadrangular; embolus system with a large, thorn-like structure (tls), a cluster of brush-like structure (bls), a broad median branch (mb) and a narrow lateral curved branch (lcb) (Fig. 2A–L).

**Female.** Unknown.

##### Distribution.

Known only from the type locality (Fig. 11).

#### ﻿ Trilacuna qingliangfeng

Taxon classificationAnimaliaAraneaeOonopidae

Ma & Tong sp. nov.

1840992B-BACC-5725-8C7F-D56B4C07AB8F

https://zoobank.org/9A23C7E9-4EB7-431B-8588-3A3DAFBB640B

**Figure 3.**
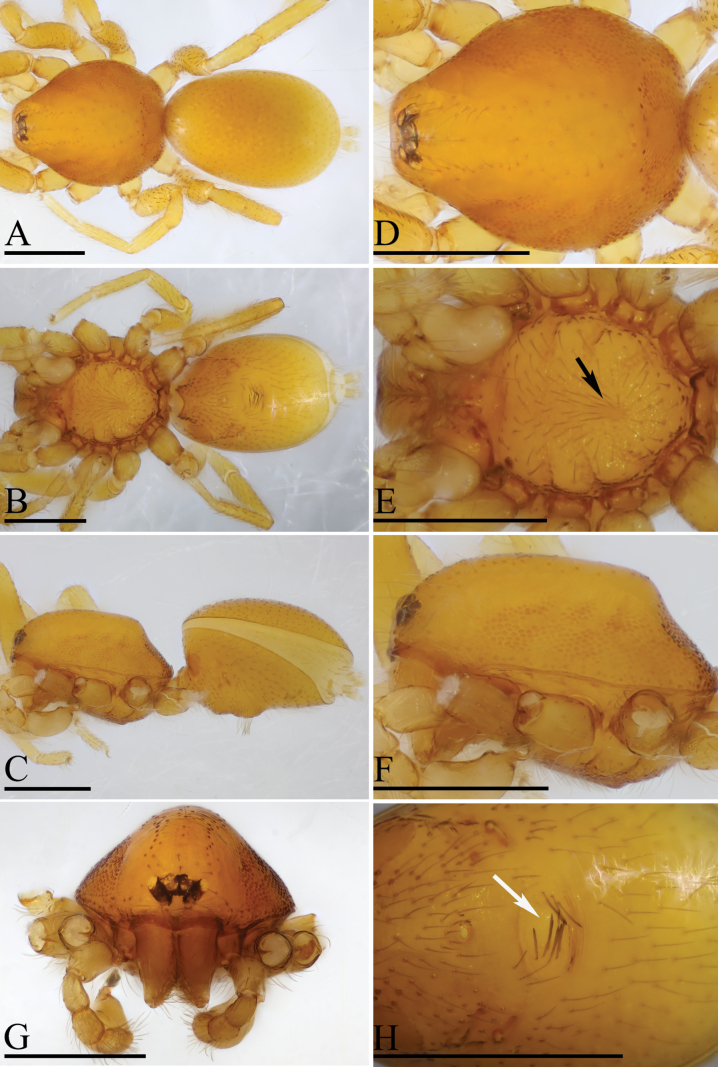
*Trilacunaqingliangfeng* sp. nov., male holotype **A–C** habitus in dorsal, ventral and lateral views **D–G** prosoma in dorsal, ventral, lateral and anterior views, arrow shows the small ridges **H** abdomen, ventral view, arrow shows the clustered setae. Scale bars: 0.4 mm (**A–H**).

**Figure 4.**
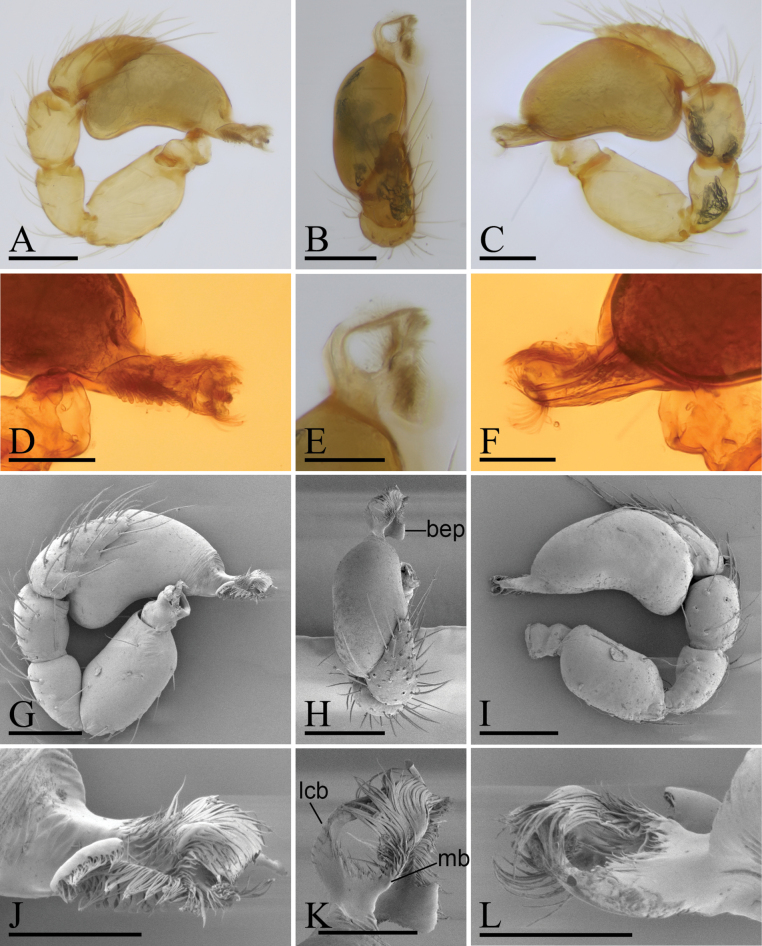
*Trilacunaqingliangfeng* sp. nov., left male palp, **A–F** (light) and **G–L** (SEM) **A, G** prolateral view **B, H** dorsal view **C, I** retrolateral view **D, J** distal part of palpal bulb, prolateral view **E, K** distal part of palpal bulb, dorsal view **F, L** distal part of palpal bulb, retrolateral view. Abbreviations: bep = basal ear-shaped projection; mb = medial branch; lcb = lateral curved branch. Scale bars: 0.1 mm (**A–C, G–I**); 0.05 mm (**D–F, J–L**).

**Figure 5.**
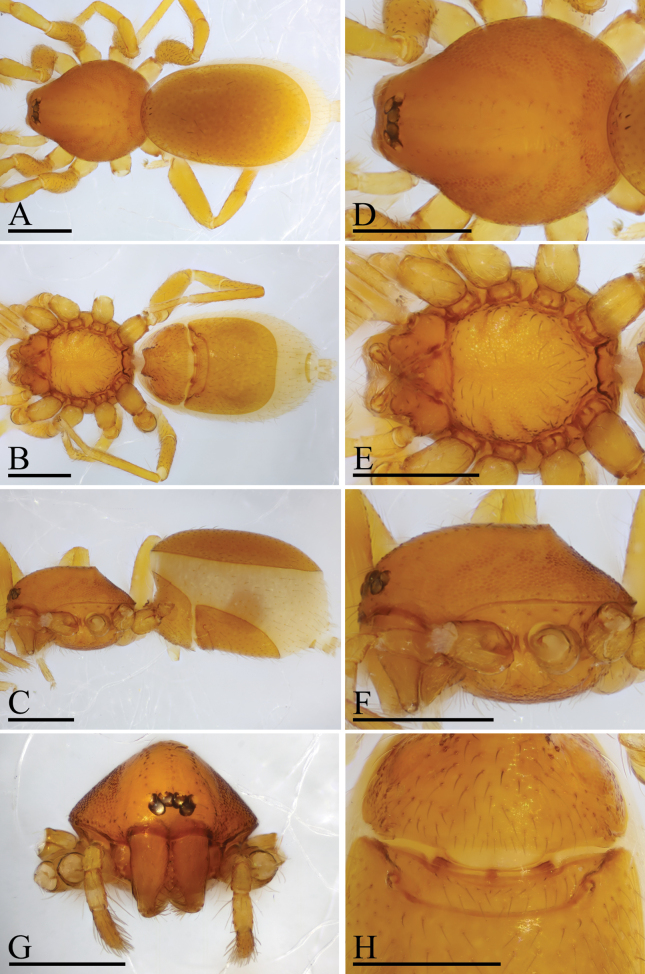
*Trilacunaqingliangfeng* sp. nov., female paratype **A–C** habitus in dorsal, ventral, and lateral views **D–G** prosoma in dorsal, ventral, lateral and anterior views **H** abdomen in ventral view. Scale bars: 0.4 mm (**A–H**).

**Figure 6.**
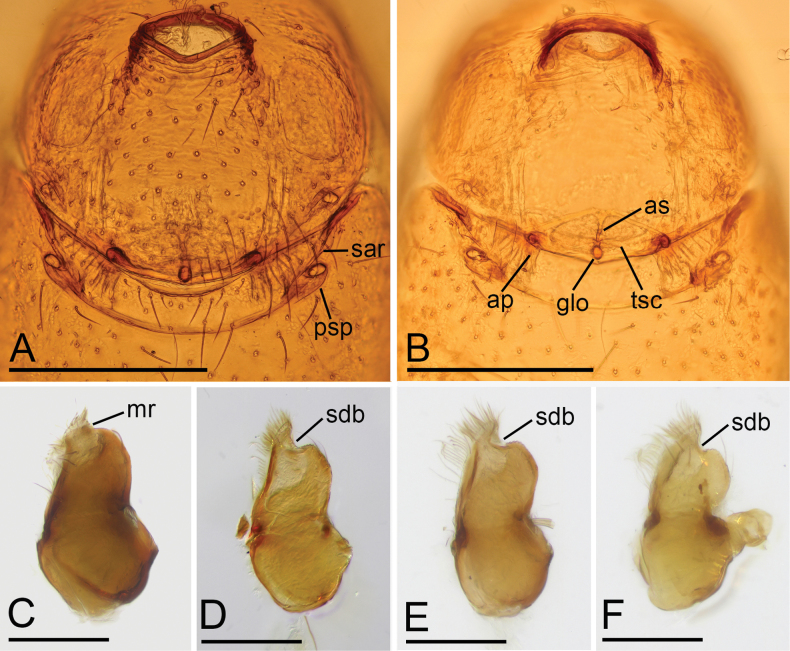
*Trilacunaqingliangfeng* sp. nov., female paratype (**A, B**), *Trilacunaguangwu* sp. nov., male holotype (**C**), *Trilacunaqingliangfeng* sp. nov., male holotype **(D)**, *Trilacunataoyuanyu* sp. nov., male holotype (**E**), *Trilacunayunmeng* sp. nov., male holotype (**F**) **A, B** copulatory organ in ventral and dorsal views **C–F** endites in ventral view. Abbreviations: ap = apodemes; as = anterior sclerite; glo = globular structure; mr = membranous region; psp = posterior spiracle; sar = sclerotized, recurved arches; sdb = slightly curved distal branch; tsc = transverse bars. Scale bars: 0.4 mm (**A, B**); 0.2 mm (**C–F**).

Figs 3 , 4 , 5 , 6A, B, D

##### Material examined.

***Holotype***: China • ♂ (SYNU-1181); Anhui Province, Xuancheng City, Jixi County, Qingliangfeng National Nature Reserve, Zhanlingwan; 30°5'53"N, 118°51'59"E; 8.VI.2014; F. Zhang leg.

***Paratypes***: China • 2 ♂ 8 ♀ (SYNU-1182–1191); same data as holotype • 4 ♂ 6 ♀ (SYNU-1207–1216); same data as holotype; 28.VIII.2014 • 1 ♂ 8 ♀ (SYNU-1217–1225); Qingliangfeng National Nature Reserve, Lantianao; 30°8'2"N, 118°49'42"E; 29.V.2014; F. Zhang leg. • 1 ♀ (SYNU-1227); Qingliangfeng National Nature Reserve, Yezhutan; 30°7'10"N, 118°51'10"E; 30.V.2014; F. Zhang leg. • 1 ♂ 9 ♀ (SYNU-1228–1237); Qingliangfeng National Nature Reserve, Fuling Town, Yonglai Village; 30°9'8"N, 118°51'3"E; 5.VI.2013; Cha and Zhang leg. • 1 ♂ 4 ♀ (SYNU-1202–1206); Qingliangfeng National Nature Reserve, Fuling Town, Yonglai Village; 30°9'8"N, 118°51'3"E; 3.VI.2013; Cha and Zhang leg. • 2 ♂ 1 ♀ (SYNU-1240–1242); Qingliangfeng National Nature Reserve, Fuling Town, Yonglai Village, Qingliangfeng Protection Station; 30°2'32"N, 118°50'27"E; 4.VI.2013; Cha and Zhang leg. • 2 ♀ (SYNU-1238–1239); Qingliangfeng National Nature Reserve, Fuling Town, Yonglai Village, Huihanggudao; 30°7'57"N, 118°48'17"E; 2.VII.2013; Cha and Zhang leg. • 3 ♀ (SYNU-1199–1201); Qingliangfeng National Nature Reserve, Shexian County, Zhupu; 29°50'51"N, 118°25'49"E; 6.VI.2013; Cha and Zhang leg.

##### Other materials.

China • 1 ♂ 3 ♀ (SYNU-536); Anhui Province, Huangshan City, Xiuning County, Qiyun Mountain; 29°48'23"N, 118°2'24"E; 19.IV.2011; Zongxu Li and Luyu Wang leg. • 1 ♂ 1 ♀ (SYNU-537); same data as above • 2 ♀ (SYNU-538); same data as above • 1 ♀ (SYNU-1226); Anhui Province, Xiuning County, Lingnan Town, Dawu; 29°25'22"N, 118°8'12"E; 4.VI.2014; F. Zhang leg. • 7 ♀ (SYNU-1192–1198); Zhejiang Province, Tianmushan Mountain, Chanyuan Temple; 30°13'50"N, 119°26'55"E; 10.VI.2014; F. Zhang leg. • 1 ♂ (SYNU-1243); Zhejiang, Tianmushan Mountain, Qianmutian; 30°23'30"N, 119°26'54"E; 10.VI.2014; F. Zhang leg.

##### Etymology.

The specific name is a noun in apposition taken from the type locality.

##### Diagnosis.

The new species is similar to *Trilacunawenfeng* Tong & Li, 2021, but can be distinguished by the slightly elevated epigastric region and a group of strong setae behind the level between posterior spiracles (Fig. 3C, H) vs strongly elevated, with a cluster of densely short setae in front of the level between posterior spiracles (Wang et al. 2021: fig. 6E, G, H), by the ridges on central area of sternum (Fig. 3E) vs lacking (Wang et al. 2021: fig. 6D), and by the small globular structure of endogyne (Fig. 6B) vs spiral-shaped (Wang et al. 2021: fig. 5D).

##### Description.

**Male** (Holotype). ***Body***: yellow, legs lighter; habitus as in Fig. 3A–C; body length 1.63. ***Carapace***: 0.78 long, 0.63 wide; sides granulate, lateral margin rebordered (Fig. 3D). ***Eyes***: nearly equal sized; posterior eye row recurved from above, procurved from front; ALE separated from edge of carapace by 1.3 diameters (Fig. 3D, G). ***Mouthparts***: chelicerae straight; labium rectangular, anterior margin deeply incised; endites slender, distally branched (Figs 3E, 6D). ***Sternum***: surface finely smooth, with several ridges on central area (Fig. 3E). ***Abdomen***: 0.91 long, 0.57 wide; booklung covers ovoid, surface smooth; sperm pore situated at level of anterior spiracles; apodemes present, posterior spiracles not connected by groove; epigastric region slightly elevated, with a group of strong setae (Fig. 3C, H). ***Leg spination***: legs I-II: tibia: v2-2-2-2-0, metatarsus: v2-2-0. ***Palp***: orange; 0.55 long (0.18, 0.11, 0.10, 0.16); femur elongated (width/length = 0.57); bulb kidney-shaped, basally strongly bulged; embolus system with ear-shaped projection (bep) at base, with a lateral curved branch (lcb) and narrow medial branch (mb) (Fig. 4A–L).

**Female** (paratype, SYNU-1184). As in male except as noted. ***Body***: habitus as in Fig. 5A–C; body length 1.98. ***Carapace***: 0.82 long, 0.66 wide. ***Mouthparts***: endites unmodified. ***Sternum***: without ridges on central area (Fig. 5E). ***Abdomen***: 1.24 long, 0.71 wide. ***Epigastric area***: with recurved, strongly sclerotized arches (sar) (Figs 5H, 6A). ***Endogyne***: with narrow, transversally elongated sclerite (tsc); with an anterior T-shaped sclerite (as) and a posterior small globular structure (glo) (Fig. 6B).

##### Distribution.

Known only from the type locality (Fig. 11).

#### ﻿ Trilacuna taoyuanyu

Taxon classificationAnimaliaAraneaeOonopidae

Ma & Tong sp. nov.

AA01F232-4EBD-5314-86C3-8C7C5B1D1214

https://zoobank.org/1264BFD5-911F-4D6F-8871-4246597B9858

Figs 6E , 7 , 8

##### Material examined.

***Holotype***: China • ♂ (SYNU-1178); Fujian Province, Wuyishan City, Wuyi Mountain Scenic Area, Taoyuanyu; 27°39'15"N, 117°56'54"E; 9.VI.2013; C. Jin leg.

##### Etymology.

The specific name is a noun in apposition taken from the type locality.

##### Diagnosis.

**Figure 7.**
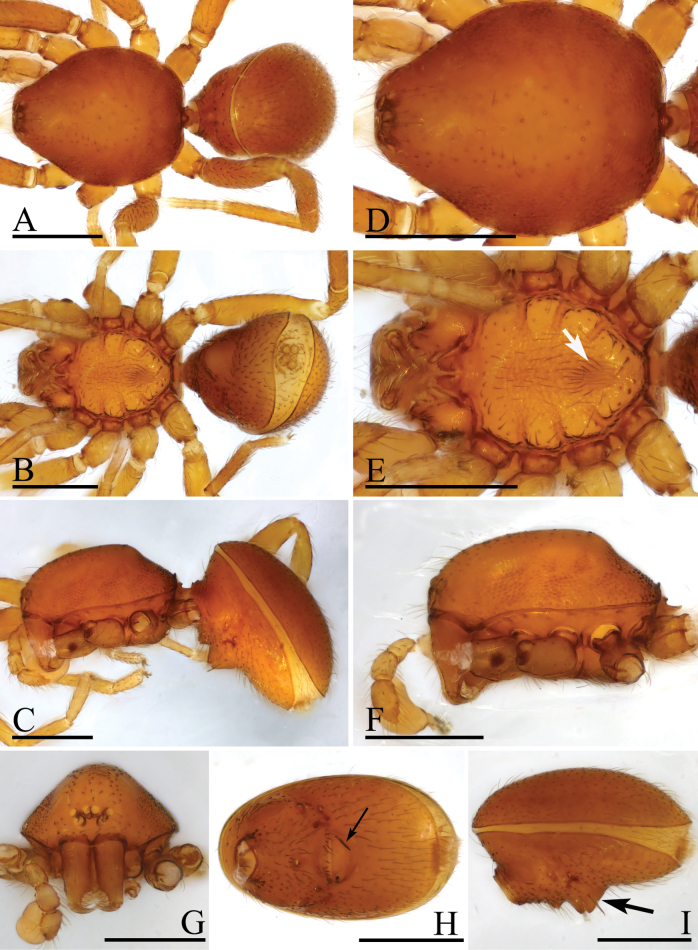
*Trilacunataoyuanyu* sp. nov., male holotype **A–C** habitus in dorsal, ventral, and lateral views **D–G** prosoma in dorsal, ventral, lateral and anterior views, white arrow shows the small ridges **H–I** abdomen, ventral and lateral views, arrow in **H** shows the strong setae, and arrow in **I** shows the strong elevated epigastric region. Scale bars: 0.4 mm (**A–I**).

**Figure 8.**
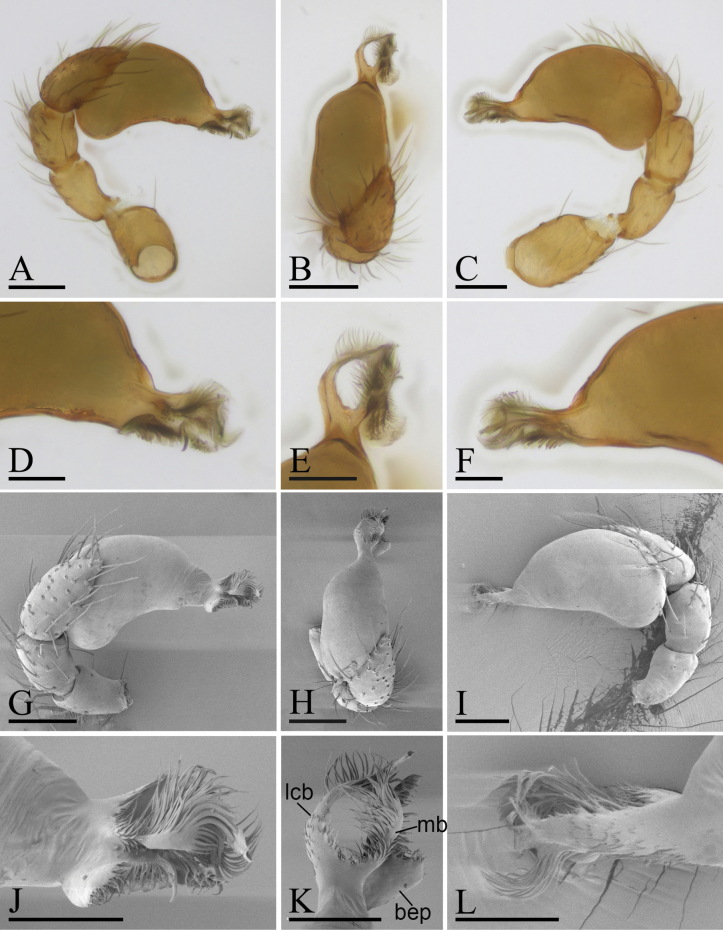
*Trilacunataoyuanyu* sp. nov., left male palp, **A–F** (light) and **G–L** (SEM) **A, G** prolateral view **B, H** dorsal view **C, I** retrolateral view **D, J** distal part of palpal bulb, prolateral view **E, K** distal part of palpal bulb, dorsal view **F, L** distal part of palpal bulb, retrolateral view. Abbreviations: bep = basal ear-shaped projection; lcb = lateral curved branch; mb = medial branch. Scale bars: 0.1 mm (**A–C, G–I**); 0.05 mm (**D–F, J–L**).

The new species is similar to *Trilacunabawan* Tong, Zhang & Li, 2019, but can be distinguished by the strongly elevated epigastric region and two strong setae on it (Fig. 7H, I), vs slightly elevated, without strong setae (Tong et al. 2019: fig. 1H, I), and the kidney-shaped palpal bulb (Fig. 8A, C) vs oval, with deeply constriction on distal region (Tong et al. 2019: figs 2A, B, 22A, B).

##### Description.

**Male** (Holotype). ***Body***: reddish brown, chelicerae and sternum lighter, legs yellow; habitus as in Fig. 7A–C; body length 1.71. ***Carapace***: 0.79 long, 0.63 wide; sides granulate, lateral margin rebordered (Fig. 7D). ***Eyes***: ALE largest, PLE and PME nearly equal sized; posterior eye row recurved from above, procurved from front; ALE separated from edge of carapace by 1.4 diameters (Fig. 7D, G). ***Mouthparts***: chelicerae straight; labium rectangular, anterior margin deeply incised; endites slender, distally branched (Figs 6E, 7E). ***Sternum***: surface rugose, with several ridges on posterior area (Fig. 7E). ***Abdomen***: 0.92 long, 0.56 wide; booklung covers ovoid, surface smooth; sperm pore situated at level of anterior spiracles; apodemes present, posterior spiracles connected by groove; epigastric region strongly elevated, with 2 very long, strong setae (Fig. 7H, I). ***Leg spination***: legs I-II: tibia: v2-2-2-2-0, metatarsus: v2-2-0. ***Palp***: orange; 0.54 long (0.19, 0.11, 0.09, 0.15); femur elongated (width/length = 0.59); bulb kidney-shaped, basally slightly bulged; embolus system with ear-shaped projection (bep) at base, with a lateral curved branch (lcb) and narrow medial branch (mb) (Fig. 8A–L).

**Female.** Unknown.

##### Distribution.

Known only from the type locality (Fig. 11).

#### ﻿ Trilacuna yunmeng

Taxon classificationAnimaliaAraneaeOonopidae

Ma & Tong sp. nov.

05BD8393-EA28-580D-B984-AFCD0C65D621

https://zoobank.org/B6941EF8-9CE0-486F-A338-2053D7607BD7

Figs 6F , 9 , 10

##### Material examined.

***Holotype***: China • ♂ (SYNU-1180); Hebei Province, Baoding City, Yi County, Yunmeng Mountain; 39°23'52"N, 115°16'17"E; 11.VI.2012; F. Zhang leg.

##### Etymology.

The specific name is a noun in apposition taken from the type locality.

##### Diagnosis.

**Figure 9.**
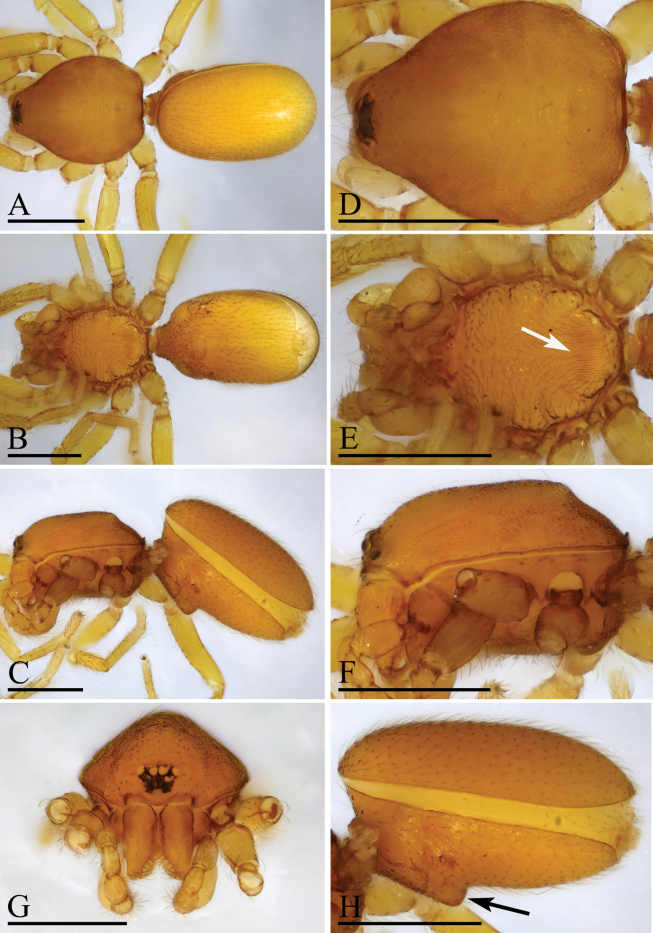
*Trilacunayunmeng* sp. nov., male holotype **A–C** habitus in dorsal, ventral and views **D–G** prosoma in dorsal, ventral, lateral and anterior views, arrow shows the small ridges **H** abdomen, lateral view, arrow shows the steeply elevated epigastric region. Scale bars: 0.4 mm (**A–H**).

The new species is similar to *Trilacunahansanensis* Seo, 2017, but can be distinguished by the many ridges on posterior area of sternum (Fig. 9E), vs a pair of ridges (Seo 2017: fig. 1B), and the steeply elevated epigastric region (Fig. 9H) vs flat (Seo 2017: fig. 1B).

##### Description.

**Figure 10.**
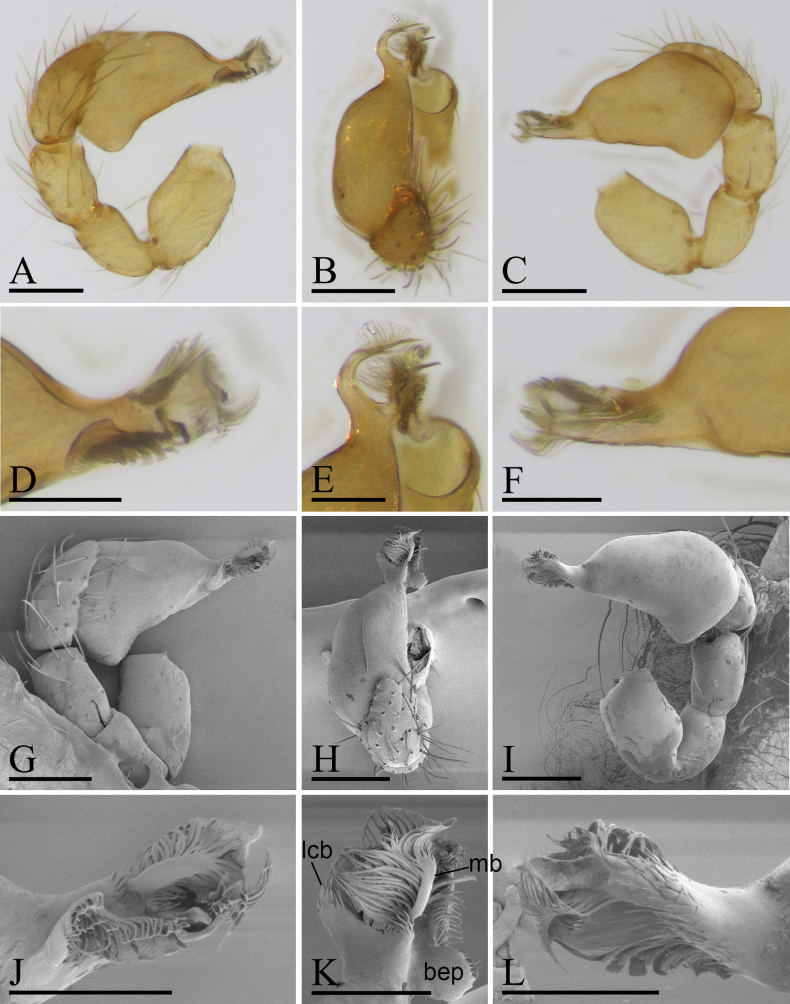
*Trilacunayunmeng* sp. nov., left male palp, **A–F** (light) and **G–L** (SEM) **A, G** prolateral view **B, H** dorsal view **C, I** retrolateral view **D, J** distal part of palpal bulb, prolateral view **E, K** distal part of palpal bulb, dorsal view **F, L** distal part of palpal bulb, retrolateral view. Abbreviations: bep = basal ear-shaped projection; lcb = lateral curved branch; mb = medial branch. Scale bars: 0.1 mm (**A–C, G–I**); 0.05 mm (**D–F, J–L**).

**Male** (Holotype). ***Body***: yellow, legs lighter; habitus as in Fig. 9A–C; body length 1.57. ***Carapace***: 0.67 long, 0.57 wide; sides granulate, lateral margin rebordered (Fig. 9D). ***Eyes***: nearly equal sized; posterior eye row recurved from above, procurved from front; ALE separated from edge of carapace by 1.3 diameters (Fig. 9D, G). ***Mouthparts***: chelicerae straight; labium rectangular, anterior margin deeply incised; endites slender, distally branched (Figs 6F, 9G). ***Sternum***: surface finely smooth, with many ridges on posterior area (Fig. 9E). ***Abdomen***: 0.84 long, 0.57 wide; booklung covers ovoid, surface smooth; sperm pore situated at level of anterior spiracles; apodemes present, posterior spiracles not connected by groove; epigastric region steeply elevated (Fig. 9C). ***Leg spination***: legs I–II: tibia: v2-2-2-2-0, metatarsus: v2-2-0. ***Palp***: orange; 0.50 long (0.14, 0.11, 0.11, 0.14); femur elongated (width/length = 0.62); bulb triangular, basally strongly bulged; embolus system with ear-shaped projection (bep) at base, with a lateral curved branch (lcb) and narrow medial branch (mb) (Fig. 10A–L).

**Female.** Unknown.

##### Distribution.

**Figure 11.**
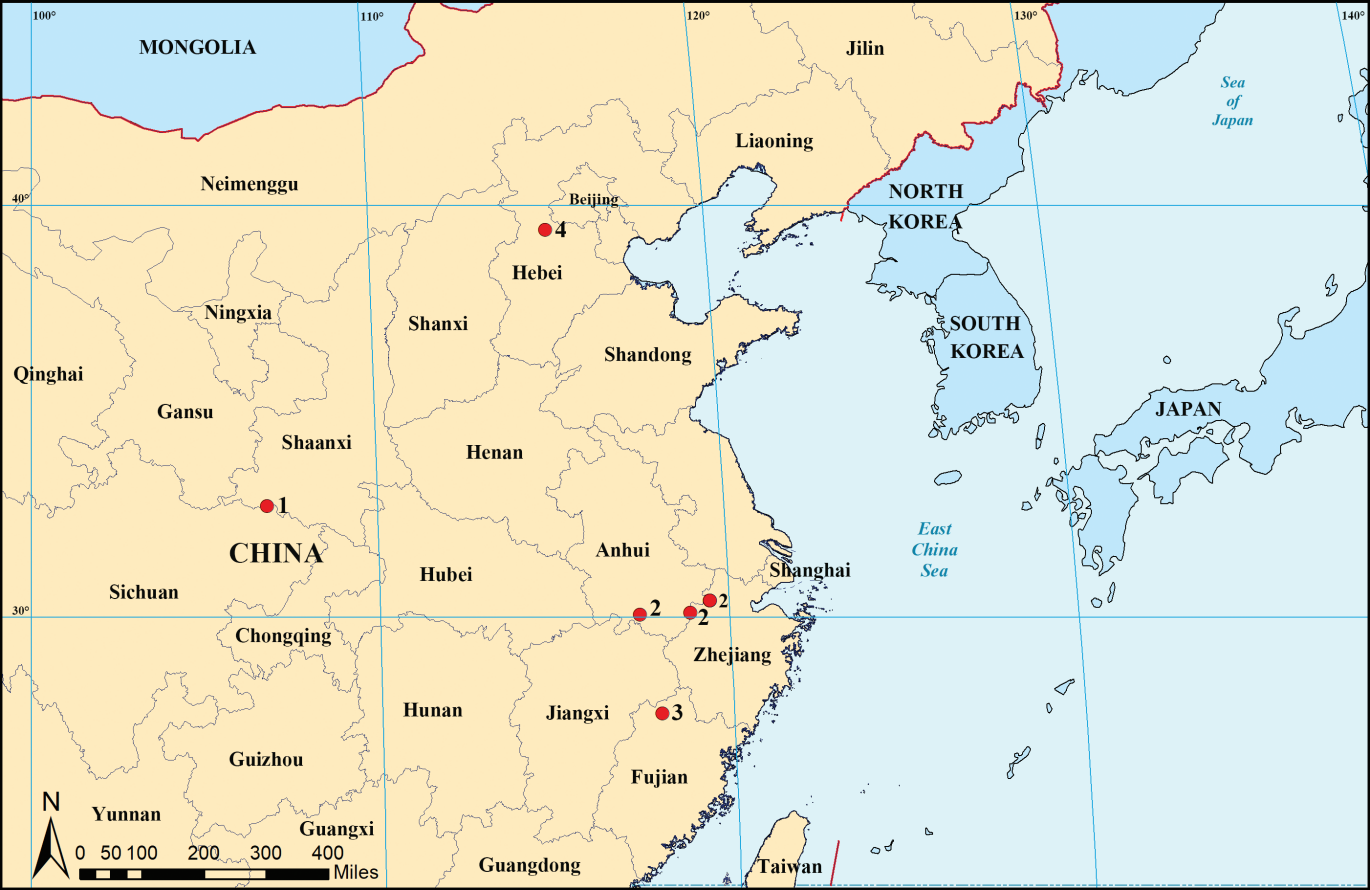
Distribution records of four new species from China. 1 = *Trilacunaguangwu* sp. nov.; 2 = *Trilacunaqingliangfeng* sp. nov.; 3 = *Trilacunataoyuanyu* sp. nov.; 4 = *Trilacunayunmeng* sp. nov.

Known only from the type locality (Fig. 11).

## Supplementary Material

XML Treatment for Trilacuna

XML Treatment for Trilacuna guangwu

XML Treatment for Trilacuna qingliangfeng

XML Treatment for Trilacuna taoyuanyu

XML Treatment for Trilacuna yunmeng

## ﻿Additional information

### Author ORCIDs

Jimeng Ma https://orcid.org/0009-0008-4579-3506

Qiang Chen https://orcid.org/0009-0007-9722-1561

Yanfeng Tong https://orcid.org/0000-0002-4348-7029

### Data availability

All of the data that support the findings of this study are available in the main text.
